# A Qualitative Exploration of Ethical Aspects of Using AI in Parkinson Disease: Patient Panel Study

**DOI:** 10.2196/74144

**Published:** 2026-04-28

**Authors:** Jamie Linnea Luckhaus, Therese Scott Duncan, Anna Kharko, Anna Clareborn, Maria Hägglund, Charlotte Blease, Sara Riggare

**Affiliations:** 1 Department of Women's and Children's Health, Participatory eHealth and Health Data, Disciplinary Domain of Medicine and Pharmacy, Uppsala University, MTC-huset, Dag Hammarskjölds väg 14B, 1 tr, Uppsala, Sweden, 46 0769434111; 2 Department of Immunology, Genetics and Pathology, Uppsala University, Uppsala, Sweden; 3 Department of Medical Sciences, Uppsala University Hospital, Uppsala, Sweden; 4 Department of Psychiatry, Beth Israel Deaconess Medical Center, Harvard University, Boston, United States; 5 Center for Disability Research, Disciplinary Domain of Medicine and Pharmacy, Uppsala University Hospital, Uppsala, Sweden

**Keywords:** artificial intelligence, AI, co-design, medical ethics, biomedical ethical principles, Parkinson disease, predictive medicine, precision medicine, user perceptions, qualitative study

## Abstract

**Background:**

As Parkinson disease (PD) rates increase, so does interest in finding new technological solutions for PD management. Despite substantial efforts to explore potential applications of artificial intelligence (AI) in PD management, research from the perspectives of people with PD on AI remains limited.

**Objective:**

This study aims to explore the ethical considerations of AI in PD management from the perspective of people with PD.

**Methods:**

A qualitative triangulation of 13 interviews and 2 focus groups (FGs) with a panel of expert-by-experience people with PD from 6 European countries was carried out using abductive thematic analysis. The 6 biomedical ethical principles conceptualized by Beauchamp and Childress guided the analysis. Participants varied in diagnosis, disease experiences, and technological backgrounds. A researcher with PD was involved from start to finish, providing valuable insights into data collection and analysis.

**Results:**

Although optimistic that AI could enhance autonomy and beneficence through personalized, actionable insights for people with PD and their health care professionals, concerns arose over patient involvement, model accuracy and privacy, ethical injustices, and the psychological impact. Risk prediction, prognosis, and medication response were viewed differently in terms of potential value and ethical considerations, with risk prediction being perceived as the most ethically complex. To uphold autonomy, it was considered important for AI insights to be patient-accessible, and sensitive insights should be communicated by a health care professional who recognizes individual differences in desiring and responding to AI predictions.

**Conclusions:**

While people with PD felt AI could personalize (self-)care and increase autonomy, concerns about psychological harm and widening inequalities highlight the importance of ethical safeguards. Our findings underscore the importance of AI integrations that prioritize individual needs, actively engage people with PD in the development, implementation, and interpretation of predictive AI, and establish guidelines to support health care professionals and minimize patient harm. Different forms of implementation and precautions should be taken for risk, progression, and medication response prediction.

## Introduction

Artificial intelligence (AI) and its promise of delivering individually tailored health care is gaining traction, especially for chronic conditions such as Parkinson disease (PD), the fastest growing neurodegenerative condition [1]. There are numerous challenges in diagnosing and managing PD, including overlapping symptoms with other diseases, the presentation of nonmotor prodromal symptoms years before cardinal symptoms, and variability in disease presentation and medication response across individuals and over the course of the disease [2,3].

Many proof-of-concept studies on PD monitoring, clinical decision-making, and diagnostic technologies through analyzing digital biomarkers have been published recently. These include AI to detect PD and predict progression [4], assess motor states over the course of a levodopa dose [5], and classify ON-OFF fluctuations [6]. The idea behind applying predictive AI, which forecasts future events or outcomes based on historical trends, is ultimately to provide more timely and individually tailored treatments than a clinician could do on their own.

Rapid eye movement sleep behavior disorder (RBD) is a possible prodromal marker of neurodegenerative diseases [7], making this patient group a target for PD risk monitoring, with hopes of early neuroprotective interventions as well as learning more about prodromal PD. However, with PD being progressive with no cure, predicting risk comes with ethical considerations such as psychological distress and stigmatization, especially given the high uncertainty of current model calculations [8,9]. Although neuroprotective therapies to slow progression are under development [10] and modifiable lifestyle factors such as exercise have also been suggested to play an important role in symptom management [11], a PD diagnosis would, for most, constitute life-altering news. A study on whether and how to disclose PD risk to patients with RBD suggests that this depends on patient subtype and must respect individual needs and wishes [9]. Literature on the development of technologies for PD highlights the importance of involving people with PD in the design process to ensure alignment with real-world problems [12,13]. Leveraging experiential knowledge through a co-design approach helps capture the psychological, social, and medical factors of the user experience, resulting in sustained adoption [13].

**Figure 1.**
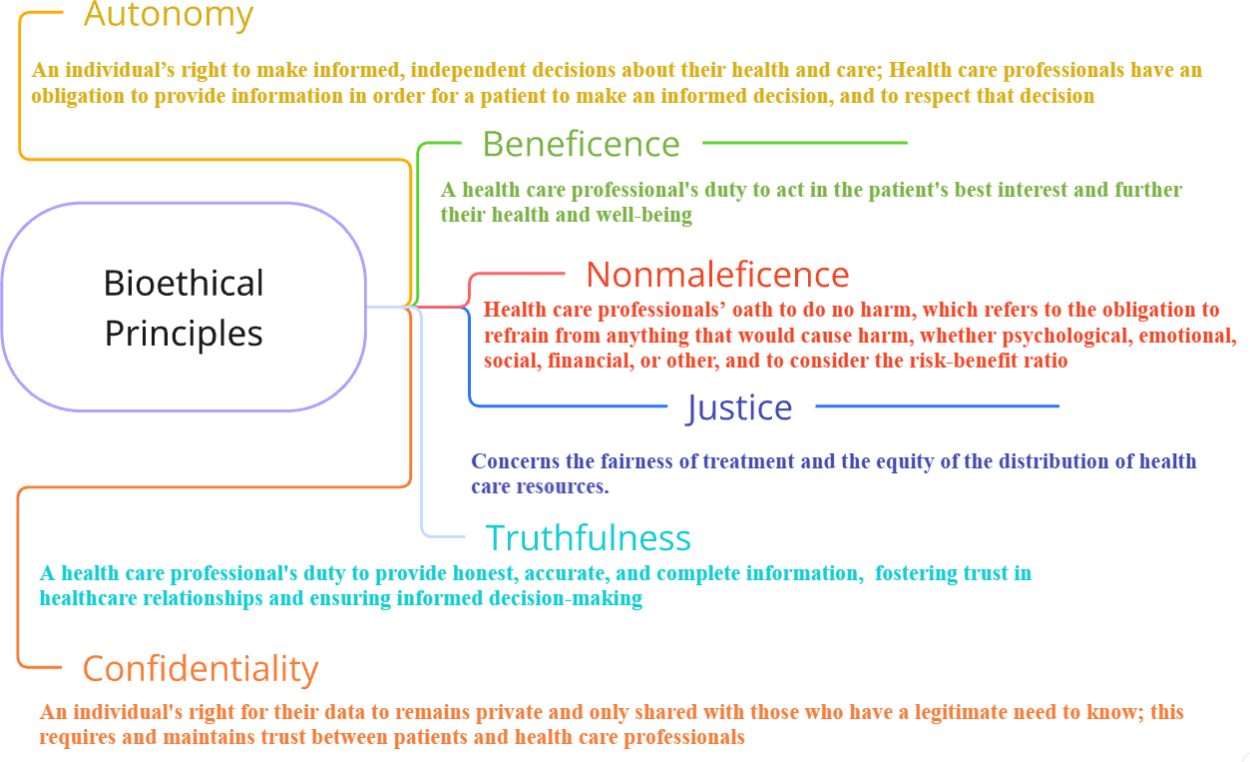
The 6 bioethical principles and their definitions (adapted from Beauchamp and Childress [15]).

The limited research on AI-assisted monitoring from the perspective of people with PD has found multilevel ethical concerns regarding AI prognostics: on personal, interpersonal, professional or institutional, and societal levels [8,14]. As AI use for PD increases, it is essential to explore the ethical dimensions from the users’ perspective, including perceptions of benefits, potential harms, privacy risks, and broader concerns. A framework that can help map ethical considerations is the bioethics framework [15] (Figure 1), which has previously been used to contextualize and evaluate the impact of digital interventions on patient autonomy, beneficence, nonmaleficence, justice, truthfulness, and confidentiality [16,17]. These considerations are crucial for the development and implementation of such tools, to increase value and avoid harm for potential users. We, therefore, aim to explore the ethical considerations of using AI in PD management from the perspective of people with PD.

## Methods

### Design and Setting

This study presents findings from qualitative data collected within the European Union (EU)–funded project: “AI-PROGNOSIS: Artificial intelligence–based Parkinson’s disease risk assessment and prognosis” (Nr.101080581). To provide a perspective of people with PD throughout the project, an international patient panel was assembled in late 2023, which served as the participant group. The panel was recruited using a combination of convenience, purposive, and snowball sampling to reach multiple English-speaking people with PD from each of the 6 countries with an appropriate background. People with PD were selected based on their high PD knowledge levels, high levels of engagement in PD communities, and interest in technology, while at the same time maintaining diversity in age, gender, country, time since diagnosis, and educational or professional background. A few participants had prior (professional) knowledge of AI. An hour-long information meeting was held online for those interested prior to officially inviting participants, and those who missed were sent the recording.

We adopted participatory and co-design methodologies, which are important in the development of digital health tools, ensuring that the technology is useful and user-centered [13,18,19]. A researcher with PD was actively involved in all phases of the study: planning and design, data collection and analysis, and writing.

The 3 models under development within AI-PROGNOSIS, which were discussed with the panel, were [1] a risk score, predicting one’s risk of developing PD among individuals clinically identified as at risk [2]; a prognosis, predicting the progression of people with PD; and [3] medication response, predicting the response of people with PD to various medications and timings or doses.

### Data Collection

The participants (patient panel) consisted of 14 people with PD (5 women and 9 men) from 6 countries: France (n=2), Germany (n=3), Portugal (n=3), Spain (n=1), Sweden (n=2), and the United Kingdom (n=3). The mean age at the time of data collection was 53.5 (SD 11.4; range 40‐75) years. The mean age at the time of PD diagnosis was 44.5 (SD 11.8; range 32‐64) years, and the mean number of years living with PD was 9 (SD 5; range 1‐17). All were of Caucasian ethnicity.

Semistructured interviews (January-March 2024) at an average of 36 minutes (range 13‐57 min) were conducted by JLL with 13 out of 14 panel members. One panel member chose not to interview but participated in an FG. The interview guide (Multimedia Appendix 1) covered four topics: (1) personal and PD background; (2) prior research participation (if any); (3) expectations, concerns, and hopes regarding this project; and (4) views on AI in health care or research in general and in AI-PROGNOSIS. No introduction to AI was provided prior to the interviews, in order to capture initial thoughts and expectations, as these were the first interviews of the project.

An introduction to the project’s AI tools and key development terms (eg, high-level features, user-needs, explainable insights) was given before each of the 2 FGs, however, to facilitate deeper and well-informed discussion (Multimedia Appendix 2). The FGs were attended by 9 or 14 panel members and were facilitated by SR and AC in February 2024. The FGs were held on 2 occasions to increase participation while keeping the size small enough for meaningful conversation, and participants signed up based on their schedule. Only the 2 most popular times were selected, meaning not all participated. Additionally, the 2 French participants joined the project after the FGs due to English language recruitment challenges. The FG objective was to elicit user needs for the project’s development and was held ahead of prototypes, so participants spoke hypothetically. For the sake of the discussion, participants were asked to respond with the assumption that the AI models would work perfectly. The content was similar between the two (the guide is provided in Multimedia Appendix 3), although the facilitators gave the second FG more time on questions unanswered by the first to ensure data on all topics of interest. FG 1 (1 hour 48 min) consisted of 2 female and 2 male panel members from 3 different countries, and FG 2 (1 hour 43 min) consisted of different participants: 3 female and 2 male panel members from 4 countries. The participants were divided with availability and diversity in mind. Each FG consisted of a primary and secondary facilitator, as well as 1 additional project member, who facilitated note-taking. All data were collected over Zoom in English, with occasional clarifications as necessary in participants’ native language to maximize participation and understanding.

### Data Analysis

**Figure 2.**
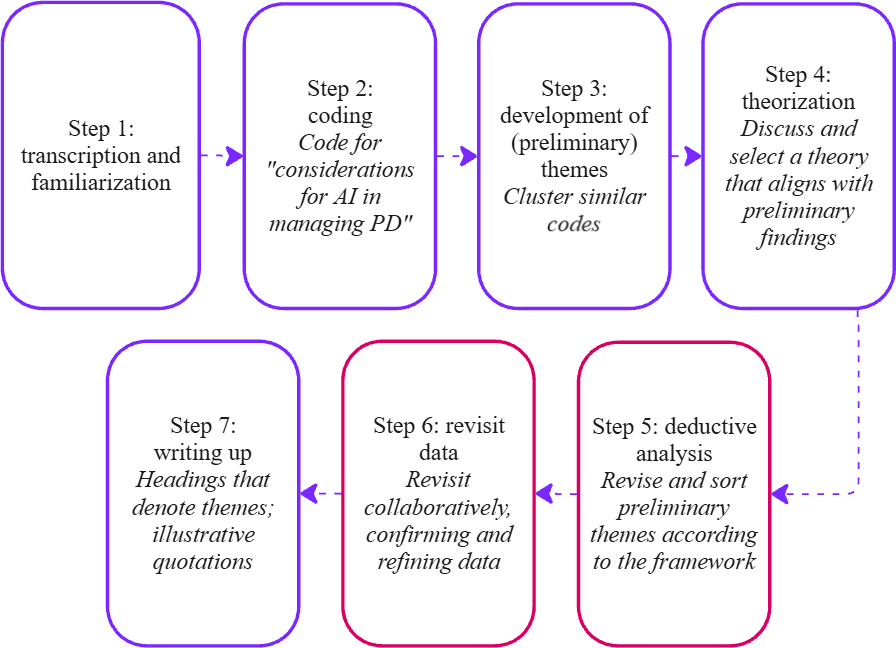
Our 7-step analysis process. Red boxes indicate differences from the 8-step abductive thematic analysis by Thompson [20]*.* AI: artificial intelligence; PD: Parkinson disease.

**Figure 3.**
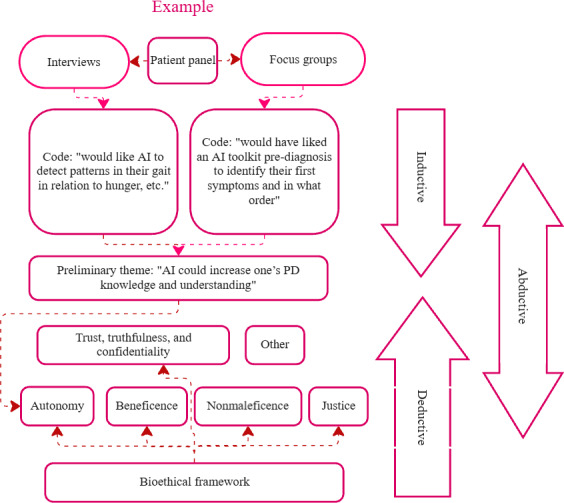
Mapping of our abductive thematic analysis approach. AI: artificial intelligence; PD: Parkinson’s disease.

The interviews and FGs were analyzed abductively as 1 dataset, using a simplified version of Thompson’s 8-step abductive thematic analysis (Figure 2) [20]. This method of abductive reasoning, shown in Figure 3, involves iteratively moving between empirical data and theoretical framework to refine interpretations [21]. JLL familiarized herself with the data by transcribing the interviews and FGs and reading the transcripts. She then conducted inductive coding in Taguette, using “considerations for AI in managing PD” as an initial analytic lens. Codes were low-level descriptors of salient statements emerging from the text. The codes were then transferred into Miro, a collaborative online “whiteboard,” where codes were inductively grouped into preliminary themes and discussed between authors. An overarching ethical dimension emerged, prompting the abductive step of refining the research focus toward ethical considerations and the application of biomedical ethical principles [15] to deductively categorize the codes or preliminary themes (Multimedia Appendix 4). One author (CB), an expert in biomedical ethics, provided critical input to ensure alignment between themes and principles. JLL and SR finalized the analysis in a reflexive process, revisiting the transcripts, discussing, and revising accordingly. The data were not constrained by the framework, and a section highlighting outliers is also included. Due to the elements of trust in both truthfulness and confidentiality, and the smaller themes within those two principles, we combined all 3 for the presentation of our findings.

### Ethical Considerations

Participants were informed about the study aims and procedures, including data collection and processing, potential risks related to participation, and their rights under the General Data Protection Regulation (GDPR). The data were de‑identified prior to analysis and reporting. Direct identifiers (e.g., names) and potentially identifying information such as age were removed. When presenting quotations, only broad demographic characteristics were retained to minimize the risk of re‑identification. Access to the raw data was restricted to the research team, and all data were stored on secure, password‑protected systems in accordance with GDPR requirements. All participants provided informed consent prior to participation. Ethical approval was obtained from the Swedish Ethical Review Authority (reference number: 2023‑05949‑01). Participants received a modest gift card as compensation for their time.

## Results

The results are presented according to the biomedical ethical principles: (1) autonomy; (2) beneficence; (3) nonmaleficence; (4) justice; and (5) trust, truthfulness, and confidentiality, followed by illustrative quotations.

### Autonomy: AI’s Role in Agency of People With PD

#### Overview Works

The overall feeling was that AI has the potential to facilitate or hinder autonomy depending on how it is implemented and adopted. Participants mostly expressed ways in which AI could increase autonomy through meaningful and personalized insights and respective actions to take, both independently and in collaboration with a clinician. However, if the tools were to be implemented as a strictly clinician-facing tool, they would reduce rather than facilitate the autonomy of people with PD.

#### AI Could Increase One’s PD Knowledge and Understanding

Participants believed AI could be leveraged to increase their knowledge of their general health and PD—a crucial part of autonomy—through assisted self-tracking. Access to greater insights into one’s PD enables people with PD to make choices that align with their values, goals, and individual needs. Those who already used digital health tools to monitor parameters such as sleep and heart rate expressed a desire to track PD-specific symptoms as well. Those who had tracked PD symptoms were interested in improved tracking and additional symptom monitoring.

*[I’d want feedback on] tremor. When do I have tremor, what severity? My gait, like do I walk faster in the morning or in the afternoon? Ideally, if possible, linked to data that I would probably have to input myself like, am I stressed or tired? Am I hungry? Did I exercise today?*

[Female, person with PD 04, FG1]

Participants hoped that AI could find patterns in signs and symptoms that they and their health care professionals (HCPs) could not. The advanced analyses of AI could help explain why fluctuations and specific symptoms occur and in relation to medication, menstrual cycle, and lifestyle.

*I recently had a couple of allergic reactions, and I had to get cortisone shots. I noticed straight after … that my tremor was much better, and I felt better overall. That’s interesting. So I googled histamine cortisone shots … it turned out that a lot of people searched for antihistamines and cortisone and Parkinson’s. So that’s just an example of the type of information that I feel is out there*

[Female, person with PD 04, interview]

To maximize value for people with PD, AI should provide personalized insights, considering variations in an individual’s characteristics (eg, their habits) and over time (eg, injury or diet changes), as well as disease characteristics. Building on self-tracking analyses, participants were interested in receiving an AI-generated prognosis, though the level of detail desired and the value attributed varied between participants. Some wanted to know a timeline and expected rate of progression to feel less in the dark about the future and to, for example, plan retirement.

*I would think that living in uncertainty, just knowing I have this and this and it’s inevitable, it will get worse, but I don't know if it’s in six months, or six years or 16 years, that to me after careful evaluation is not a good scenario.*

[Male, person with PD 03, FG1]

As one of the AI tools exemplified within the project was for PD risk prediction, participants reflected on predating PD symptoms and diagnosis. Between them, they described receiving their PD diagnosis anywhere from 2 months to 3 years after first seeking care for symptoms, which most felt was on the faster side for PD. However, some participants felt that AI has the potential to shorten this time of uncertainty. A participant who first received anxiety medication (without relief) for his PD symptoms expressed that “... if I had directed my attention to the symptoms at that time, and if there were an app that could identify the symptoms, I think it would have been faster to identify the disease*”* (male, person with PD 02, FGD1). Several participants reflected that, as with a prognosis, they would have liked to have found out sooner that they had PD, as it would have allowed them to prepare:

*Those two aspects, the possibilities of getting the disease, and predicting the rate of progression, I think that’s very important. It’s very important for us with Parkinson’s to have an idea, even in order to then prepare the future.*

[Male, person with PD 02, interview]

#### AI Results Must Be Actionable

Participants wanted more information about their PD patterns and prognosis, so long as it was *actionable*, whether that be preparing mentally or changing lifestyle practices. It was mentioned that many of the prodromal symptoms could easily be alleviated if detected and provided with feedback, such as “*constipation, handwriting, carrying cups of tea ... eating soup with a spoon, you know, practical things*” (Female, person with PD 11, FG1). There was interest in AI-generated trajectories demonstrating pathways based on current lifestyle factors and alternative pathways based on assumed changes.

*If AI can tell us specifically that if I do some things, then in 2-3 years, I’ll be at this point, and if I don’t, I’ll be at that point, I think that will be very motivating to help people change attitudes to the treatments of the disease.*

[Male, person with PD 02, interview]

Although opinions were divided, some people with PD felt that a risk score or disease prediction could motivate disease-altering behavior change, such as diet and exercise.

*… predicting the diagnosis can be very helpful if it helps people to change their behavior, because they know that they’re in the race to get the disease. And if we think well, before the first symptoms, we already had the disease 10, 15, 20 years before.*

[Male, person with PD 02, interview]

A participant with genetic PD wondered whether, had she been provided with AI-assisted monitoring, she and her physician could have identified her prodromal symptoms more easily and received lifestyle advice to delay or even prevent PD onset.

*My mother has Parkinson’s. I’ve had low blood pressure all my life, which is also a symptom. I was constantly tired for years. And if the doctor had had a bit of knowledge of Parkinson’s, maybe she could have said, “you know, you have some risk. Maybe you can take a genetic test” … if I’d known that I had a genetic mutation, maybe I could have started exercising often as a prevention of the motor symptoms or delayed the motor symptoms … or taken better care of myself the moment I started to feel the first symptoms.*

[Female, person with PD 08, interview]

It was noted that one must be “very healthy mentally” to become motivated rather than worried. Some participants felt that knowing one’s prognosis would do little or nothing for autonomy, given that PD has no cure.

#### Sharing AI-Generated Data

The potential applications and benefits of AI in health care are fundamentally dependent on ensuring that the data being collected are accessible to the end users. Participants emphasized their right to be informed about what aspects of their health AI systems are tracking, as well as the necessity of data interpretability to maintain autonomy in their care. If such data were withheld from patients and exclusively provided to clinicians, it would undermine the ability of people with PD to actively engage in the interpretation and management of their condition. Furthermore, the presentation of data in an overly complex or opaque manner would similarly impede patient autonomy by limiting their capacity to make informed decisions about their care.

*Facilitator: ...would it make a difference if the information was presented only to the doctor? Or if it was presented to the user themselves?*

*Person with PD (male): I would like it to be presented to me first, together with a really well-thought-out analysis of what this means and what the potential developments are … I’m doing all these tests and I know that the results are sent to my doctor, and that might not return to me. Everything might be misinterpreted or something. I would like to have control over that.*

To enhance interpretability, participants preferred trends and visualizations over raw numerical data. Beyond fostering greater autonomy in managing their PD, they emphasized that AI should be designed to promote a collaborative partnership between HCPs and people with PD. AI-assisted monitoring could extend insights beyond sporadic clinical visits, capturing meaningful data in a structured format. This, in turn, could enable more focused and relevant discussions during consultations, ensuring that both parties can prioritize what truly matters in patient care.

#### Limitations of Greater Information

Participants expressed some nuanced opinions about the limitations of autonomy; however, this was especially true for individuals without symptoms. Individuals who perceive themselves as healthy may see little value in assessing their risk for a chronic illness.

*Are [people at risk] going to want to know if they are sick anyway? We have an audience who have Parkinson’s, who are not interested at all in investigating what’s going to happen to them. And now we’re talking about a client population who haven’t even gotten it yet. So, we are searching for a statement of needs and wants for a community that would find it very hard themselves to set the parameters for this project.*

[Male, person with PD 10, FG2]

Additionally, the addictive nature of digital gadgets and personalized summaries may cost some people autonomy:

*people with Apple watches in their life, they’re all obsessed about, “Oh, look, my watch told me I slept really well last night.” It’s like, “don’t you know that yourself?”*

[Female, person with PD 04, FG1]

It was also noted that the participants, “by virtue of us being on this call,” were willing and able to participate in a panel and are likely *“*the cohort that wants to implement things and make it the best journey” (female, person with PD 11, FG1). However, such AI tools and methods would likely not be equally valuable for all people with PD.

### Beneficence: Maximizing AI’s Positive Impact

Participants identified multiple ways in which AI could offer benefits to their health, particularly by improving data collection, optimizing clinical care, and enabling early detection and intervention.

#### AI Could Optimize Data Collection and Analysis

In terms of data collection and analysis methods, participants tended to express that AI would be necessary to advance the understanding of their condition. Participants highlighted that AI tools enable automated, continuous data collection and can analyze vast datasets, reducing the burden on both patients and HCPs.

*… you have lots of data about symptoms and side effects and … so on … something that is very hard for individual people to take in, analysis, but … that’s exactly what machine learning is good at, both detecting patterns in complex sets of data … but also in using this data continuously, so that you can predict what is going to happen … which should be very important information for the healthcare sector*...

[Male, person with PD 03, interview]

AI’s ability to detect patterns and correlations beyond human capability is seen as a key advantage—for example, synthesizing anecdotal online information or scanning extensive patient records to uncover insights. By capturing and analyzing large datasets, AI can unlock valuable real-world data that traditional in-clinic assessments often overlook, transforming previously “trapped” information into actionable knowledge.

#### AI Could Support HCPs With Improved and Timely Care

Participants felt AI could enhance beneficence by helping HCPs make better-informed decisions and personalizing PD treatment. Longitudinal monitoring and predictive-AI would provide additional data and thus insights for HCPs between the short, infrequent visits. PD care has long followed the same “gold standard,” and AI could enable more personalized and efficient (self-)care decisions, something participants felt was long overdue. Such insights could improve medication adjustments, detect emerging symptoms, avoid complications, and optimize interventions such as deep brain stimulation tuning and treatment for comorbidities. AI-observed trends could be triangulated with the HCPs’ observations for a more holistic and accurate assessment, especially since PD can be observed differently even between 2 neurologists, participants noted: *“*[AI] is a tool. I don’t think it should be used to substitute doctors and people, but can be used in a clever way” (female, person with PD 08, interview).

Participants stressed that AI could never replace the value of a human but could reduce the number of visits and automate or eliminate parts of HCPs’ work. Participants also expressed hope that monitoring could facilitate early detection of new symptoms and medication side effects, such as dyskinesia, to allow early intervention and avoid certain symptoms and side effects. Therefore, although not viewed as attractive for persons at risk, participants felt a risk score might be valuable for HCPs to monitor relatives of people with genetic PD and intervene when necessary.

*[AI]’s not giving you a diagnosis; it’s giving you a risk score … a warning that you should be aware of that. So, whether that comes from an app, or a conference or a doctor … It’s just telling you to be careful because you have a high risk of bearing the disease. It’s not telling you you're going to have the disease*.

[Male, person with PD 02, FG1]

Another provocative suggestion was to use AI to predict an individual’s psychological response to an AI-generated risk score—an idea that paradoxically both acknowledges AI’s risks and expresses trust in its ability to mitigate them.

### Nonmaleficence: “Do No Harm”

#### Overview

A major ethical concern of using predictive AI for PD was perceived to be psychological harm, especially regarding risk prediction. Participants emphasized that HCPs must carefully balance potential harms against anticipated benefits when integrating AI into PD care. They underscored the importance of involving people with PD in the design phase to mitigate risks and minimize potential harm. Ensuring patient participation in AI development was seen as crucial for aligning technological advancements with patient needs, thereby enhancing both safety and usability.

#### Potential Harm of Knowing

Participants worried that receiving a negative prognosis, or (especially for someone who was otherwise healthy) receiving a high-risk score, could carry grave consequences. There was a fear of causing depression or anxiety, and an early diagnosis might do more harm than good, given that PD is incurable.

*Do you really want to know that it might be PD at this stage? Perhaps not. You should think about how you communicate [AI-generated predictions]. What would be the benefit if you could identify Parkinson’s or some other neurological disorder at a very early stage?*

[Male, person with PD 03, FGD1]

Reflecting on their own diagnosis experiences, which were with a human HCP who could still deliver information better than a digital tool, participants stressed that one’s reaction to such news is hard to predict.

*I’m highly concerned over the thought of an app presenting somebody with the news, “Oh, congratulations, you probably will develop Parkinson’s,” when I remember how I felt when the doctor said … “I’m so sorry I was so direct, you seemed very calm and able to process the information.” I was like, I’m totally calm until somebody tells me I have a life-changing disease, you know?*

[Female, person with PD 04, FG1]

Participants hoped for future research advancements leading to improved treatment options and maybe even a cure, at which point knowing one’s risk would be worth the stress. However, as little or no action can be taken at this point, several felt that, currently, the harm of knowing outweighs the benefit.

*A friend of mine, who I hadn’t spoken to in a few years asked me, “So what’s your prognosis? How are you going to end up in 10 years?” I had no idea. Nobody can tell me. Maybe that’s a good thing.*

[Female, person with PD 04, interview]

Another participant reflected: “[Those] who might be more motivated [to monitor risk] are people who know people with Parkinson’s, who would equally be more terrified of finding out they’ve got it” (female, person with PD 11, FG1). Others worried that widespread AI-based predictions might contribute to increased medical anxiety or hypochondria, driving unnecessary health care utilization. “I think [AI-generated risk prediction] might attract the wrong sort of people ... the people who always think that they have everything, they might be the ones using it, but not the ones you really want to target” (female, person with PD 09, FGD2).

Beyond psychological harm, participants raised concerns about the broader social and financial consequences of AI-driven risk prediction. A risk score could influence insurance policies, employment opportunities, or retirement plans, potentially leading to discrimination or financial instability. Participants had previously found benefits in monitoring and self-tracking but also reported experiencing the burden this can pose: “Anything you have to do every day, even if it only takes one minute, it interrupts your daily routine” (male, person with PD 03, FG1). Time and motivation vary by individual and even over time for the same individual. This also applies to knowing AI predictions; some want to know everything about their health, while others are stressed by it. Individual and situational characteristics must be weighed in each case so as not to cause more harm than good when implementing predictive AI. Passive data collection, such as through a smartwatch or monitoring of mobile typing, was mentioned as positive means of lessening this burden while also collecting the desired data.

#### Importance of Guidelines for Implementation

Participants stressed that diagnoses or risk scores should only be delivered by trained professionals, never directly by an app: “We all hear these horror stories from diagnosis meetings with your doctor, or how they treat you” (male, person with PD 03, FG1). Some had experienced distressing diagnostic encounters, such as being told their symptoms could indicate either brain cancer or PD, with no clarification until after the weekend. This raised concerns that AI-driven predictive models might similarly communicate results in an alarming way. Participants also questioned the accuracy of AI predictions, emphasizing that specialists should assess and contextualize results before disclosure. To prevent harm, clear guidelines must be established on delivering sensitive, life-altering information. As one participant put it: “You just don’t know how people are going to react. Ideally, this should always be done by a trained human professional” (female, person with PD 04, FG1). Guidelines for HCPs when delivering such results “should have been done a long time ago.”

### Justice: Fair and Equitable Use of AI

While participants felt they could potentially benefit from predictive AI and personalized monitoring, they also expressed concerns that not everyone has equal access to the resources necessary to implement AI-generated recommendations.

*I’m convinced the reason I’m doing semi-okay after 12 years [with PD] is, among other things, lifestyle, exercise, nutrition, and lack of stress. But I’m also acutely aware that that’s not a solution for a young father with three kids and a full-time job, or somebody living in Uganda, or, yeah, most of this plan is in fact, saying I do yoga every morning and try to cut down on stress. It’s very much a first-world solution to the problem. Though, finding universally applicable ways of slowing progression would be a personal priority of mine*.

[Female, person with PD 04, interview]

Tools such as predictive AI, it was perceived, could further increase inequalities with regard to autonomy and beneficence among different individuals in PD care. Another question of fairness was related to the resources that AI requires and whether the added benefit of such a toolkit as this would outweigh the cost.

*If [patients] don’t want to know, well, then that’s their personal choice. But I think if they don’t want to know anyways, then there might be no use in using AI and wasting resources for someone who doesn’t want to know*.

[Female, person with PD 09, interview]

### Trust, Truthfulness, and Confidentiality: Transparency and Reliability of AI

Privacy and confidentiality were key concerns among participants, who emphasized the need for control over their own data, including the ability to choose who can access their health information—whether care partners, HCPs, or third parties: “I live in Germany where people are, I would go so far as to say, obsessed about data protection” (female, person with PD 09 FG1). Participants stressed the need for transparency in data collection, storage, and sharing practices, ensuring that personal health data remain protected and used only with explicit consent.

*I have a genetic mutation. When I got my results, my doctor put it in a sealed envelope and said to save it very carefully, “this is the most important information you can have.” And I actually didn’t even think too much about it. But I think for a lot of people, genetic information is important. It’s extremely important and private. I think it would be important for [predictive AI] to know what kind of mutation I have, but I would also be afraid that others would know it or would misuse it.*

[Female, person with PD 08, FG2]

Building trust in AI-driven PD tools requires both transparency and user control. Participants were hesitant about whether neurologists could accurately interpret their data without their input. They emphasized that people with PD should have access to their data first, before any report is sent to HCPs, ensuring transparency and patient involvement in interpretation. Participants questioned how accurate or “truthful” AI predictions could be, given the extreme complexity and variability of PD, although they were still interested in the potential insights AI could provide. “I would like to know as much [of the AI-predictions] as possible, but I also want to know the accuracy of those predictions” (male, person with PD 03, FG1). Another participant felt confident enough in her disease stability to be interested in an AI-generated prognosis:

*I personally would like to know ... [though] I’m feeling more secure in my diagnosis seven years down the line, having seen how I’ve used the PRO-PD score, and I’ve been monitoring and I’m staying relatively stable, and I’m happy with it*

[Female, person with PD 11, FG1]

To increase trust and perceived value, participants wanted clear explanations of how the AI models work, their limitations, and their accuracy rates so as to facilitate interpretation rather than misleading.

*When we deploy [these AI tools], it is really important to emphasize how the experience of every single user is included, this is one of the pillars of AI … Because once you realize that people’s experiences are taken into account, then people will trust it for sure*.

[Male, person with PD 12, interview]

### Additional Reflections

Beyond bioethical concerns, participants highlighted practical challenges, such as cultural differences across the EU, which could complicate AI implementation:

*... the intellectual part of this challenge is how do you construct a model of care that it can dovetail with well, sometimes absolutely, utterly different [healthcare systems] and different resourcing situations?*

[Male, person with PD 10, interview]

## Discussion

### Principal Findings

Participants expressed both potential benefits and ethical concerns of implementing AI in PD management. While optimistic that AI could enhance autonomy and beneficence by capturing one’s unique health patterns, motivating behavior change, and enabling shared decision-making, concerns arose over patient involvement, privacy, and the psychological impact of predictions. Risk prediction, PD prognosis, and medication response prediction were perceived differently in terms of value and ethical considerations, with risk prediction seen as the most ethically controversial, given that people with PD often have come to terms with their diagnosis in a way that those at risk would not have. Participants cautioned that AI could widen health care disparities and emphasized the need for transparency, user control, and the involvement of people with PD in development to ensure ethical implementation.

Consistent with others’ findings, participants felt that longitudinal monitoring and predictive AI have the potential to improve understanding and communication for both people with PD and HCPs, ultimately leading to more personalized and appropriate (self-)care decisions [14]. However, participants worried about inconsistencies in the interpretation of data between people with PD and HCPs, with one participant suggesting to see and approve AI predictions before sharing them with their HCP.

Participants, in alignment with previous research [22], were interested in AI solutions that provide actionable insights, as this increases autonomy over one’s health. A review of technologies within PD cites multiple examples of how providing feedback from the digital monitoring elicited positive outcomes for the people with PD, compared to when the information was exclusively for HCPs [12]. This becomes more complex, however, with the addition of predictions. Participants were interested in AI-generated disease pathways and alternatives based on interventions, although not without skepticism. As others discuss, this may provide more false hope than benefit, given that there are currently no disease-modifying treatments, and even if such therapies become available, it may take many years to be individually tailored, given the heterogeneity of PD [22]. This leads to concerns such as people with PD feeling like a failure after complying with an intervention without any noticeable difference. In addition to symptom- and disease-altering recommendations, a motivation for predictive AI noted in our study and by others was that having a better idea of one’s future could allow them to prepare mentally and logistically (eg, retirement) [22]. Given the gravity of personal predictions and the uncertainty of model accuracy, transparency in terms of what defines a given prediction or recommended intervention and the accuracy of the models is crucial, to allow people with PD to interpret the information**—**with the help of their HCP. AI medication response prediction was viewed as the least ethically complicated and could allow participants together with their HCPs to optimize not only which medication to prescribe but also how to maximize its effects by identifying patterns.

Schaeffer et al [9] surveyed experts on whether to disclose PD risk and how (ie, as a percentage/value or using general terms); however, research on this topic from the patient perspective is limited. Our study indicated that graphs and trends were preferred over raw numbers, which were not seen as interpretable or actionable. Consistent with our findings, the experts surveyed cautioned that risk predictions could likely result in psychological distress, especially when there are questions about the accuracy of the prediction [9]. In alignment with previous findings, our participants stressed the importance of receiving AI insights from a trained HCP who can help interpret and guide them [8,22].

Participants alluded to the subject of overdiagnosis as potential outcomes of AI-assisted PD risk screening. Defined as “making people patients unnecessarily,” overdiagnosis involves a diagnosis that meets all diagnostic criteria (not misdiagnosis) but causes the patient more harm than benefit [23]. A type of overdiagnosis is overdetection*,* or the identification of abnormalities that resolve themselves, never progress into something harmful, or else progress slowly enough that they never cause harm during a person’s remaining lifetime [23]. This question regarding the value of early detection, in this case of prodromal PD, when symptoms may be minimal or unnoticed and the prognosis of the complex disease is uncertain, shows the relevance of the “right *not* to know.”

While participants stressed their “right to know” any analyses of their health data, they also highlighted the potential psychological damage and other unintentional consequences that could arise, and hence the “right *not* to know.” This ethical dilemma is discussed by Schaeffer et al [9] in the context of disclosing PD risk to patients with RBD, in which they state that “there is not only a right to know, there is also the ‘right *not* to know.’” Davies [24] outlines 2 main arguments for the “right *not* to know” as to respect autonomy and avoid harm; when a diagnosis can lead to distress and social stigma, and when there is no effective cure, the diagnosis may not be worth it. However, in a response to Schaeffer et al, Karagianis [25] points out that in order to consent to not knowing, one must first understand that risk (in this case, a patient with RBD knowing their heightened risk for neurodegenerative disease) and consent to being monitored but without knowing any resulting risk scores. Davies [24] highlights an additional complexity: because PD can be genetically inherited, learning one’s own genetic information may inadvertently disclose information about family members who have not consented to receive it. Whether to disclose such information can become a moral burden for the patient receiving the information. An interesting finding was that a participant wondered if AI could be used to predict how someone might react to an AI health prediction, essentially using the problem as the solution.

### Future Research and Implications

There is no question that AI is an increasingly important topic in health care. The 2024 Revision to the Declaration of Helsinki for ethical medical research [26] is a reflection of this trend and provides concrete focus areas that directly shape future research priorities for AI in medicine, including improved AI literacy and clarity regarding the risks of current and future AI applications. These international principles have direct implications for the use of AI in PD. For instance, the declaration’s emphasis on protecting participant data and establishing ethical data governance speaks directly to the primary concerns identified in AI health monitoring for PD, such as privacy, data ownership, and the potential for stigmatization or discrimination based on AI-generated health predictions. Future research must therefore prioritize creating frameworks that address who controls the vast amounts of personal health data collected by monitoring systems and how the data can be used without harming the individual’s social and economic well-being. Furthermore, the revised declaration highlights the need for transparency and accountability, which aligns with the FUTURE-AI framework’s principles of traceability and explainability. This is critical in PD, where disclosing information about future disease risk is already fraught with ethical complexity due to prognostic uncertainty and the lack of disease-modifying therapies. The FUTURE-AI Framework, developed by interdisciplinary experts from 50 countries to ensure trustworthy and ethical implementation of AI in health care, warns of many of the same ethical concerns, such as privacy, transparency, potential patient harm, and driving inequities. The framework provides 6 guiding principles: fairness, universality, traceability, usability, robustness, and explainability to guide best practice for the development and implementation of AI in health care [27]. This is especially important, as the increased adoption of AI could likely introduce new challenges and responsibilities for HCPs, in addition to avoiding patient harm.

Additionally, it is imperative that future research explores how these ethical considerations are influenced by diverse global contexts, including different health care systems, cultural attitudes toward technology, and varying levels of health care access. The ethical frameworks guiding AI development, such as the Declaration and the FUTURE-AI framework, are intended to be international. However, their practical application will inevitably vary.

Furthermore, AI-assisted decision-making raises questions about accountability; while human error is an expected risk in clinical practice, it is unclear if AI-driven errors are expected or acceptable. For example, it remains unclear to what extent HCPs should be held responsible for mistakes arising from AI-driven recommendations. Despite these challenges, AI could offer valuable insights into how people with PD are doing between the infrequent and brief clinical visits and foster self-tracking and self-care for a more collaborative approach to care.

### Strengths and Limitations

There are strengths and limitations to consider when interpreting the results. The panel’s reflections were based on hypotheticals, and ahead of a prototype, making them more general. People with PD discussed the risk prediction tools in the context of their diagnosis journey, which may impact their perspective, but this also mitigates the ethical concerns of asking and worrying people actually at risk. Another challenging but interesting aspect of this study is that it focused on estimating PD risk and prognosis as well as medication response for people with PD. This made it at times difficult to separate which tools and predictions participants referred to.

We did not explicitly ask about ethics in either form of data collection, so it is possible that the participants could have additional thoughts on ethical considerations. The topic, instead, emerged naturally among the participants, which is also a strength, as it was unsolicited, highlighting the importance of ethics in such a context. Another consideration is that the data included questions centered around the AI-PROGNOSIS project specifically, as well as about AI in health care and research in general. This was considered in the analysis process, and a project example was in many cases useful to structure and focus the conversation. In some cases, participants spoke of all AI as one, making it unclear as to whether they were referencing predictive or perhaps generative AI, which may reflect the “black box” behind AI and indicate a need for user education when implementing such tools.

It is a strength that our participants represent multiple European countries; however, there are limitations to consider regarding the transferability of our findings. Paccoud et al [28] found that user preferences varied across EU countries and clinical and sociodemographic factors, though our participants did not mention many cultural factors. Our participants, being on an expert panel, likely differ from the average people with PD in many ways, given that they are well educated, quite involved in their own PD management, as well as PD awareness or advocacy. Many were also quite tech-savvy and had self-tracked in some manner prior, suggesting that this group might be more open toward AI but might also have more specific and well-thought-out feedback. Additionally, several of our participants have young-onset PD, and the mean age at the time of this study was 53.5 (SD 11.4), younger than the average people with PD. Older or cognitively impaired people with PD may encounter more barriers to navigating and interpreting the AI feedback and may not engage with the technology as much as our participants wish to. A recent survey on willingness to use digital medical devices among people with PD in Europe found that willingness decreased with age [28]. Another study found a strong significant correlation between cognitive ability and perceived ability to use everyday technology among people with PD [29]. At the same time, it may be considered a strength that our study provides insights into the ethical considerations of disclosing risk and progression to middle-aged individuals without cognitive impairment, some of whom are still working, for whom a prediction of disease progression may represent a more pronounced shock or harsher news.

Finally, given that our participants were Caucasian and from high-income countries, their experiences and perspectives may be very different from those from minority groups and of low- and middle-income countries, as one of our participants pointed out.

### Conclusions

Our findings highlight the need to balance hope and harm in the application of AI in PD management. We outline potential benefits for people with and without PD, HCPs, and research, as well as important concerns from the perspective of people with PD. Involving people with PD in development going forward is crucial for aligning technological advancements with patient needs and ensuring the successful and ethical adoption of AI in PD care.

## Supplementary material

10.2196/74144 Multimedia Appendix 1 Interview guide.

10.2196/74144 Multimedia Appendix 2 Focus group introduction slides.

10.2196/74144 Multimedia Appendix 3 Focus group guide.

10.2196/74144 Multimedia Appendix 4 Demonstration of analysis process.

## Abbreviations

AI: artificial intelligence
EU: European Union
FG: focus group
HCP: health care professional
PD: Parkinson disease
RBD: rapid eye movement sleep behavior disorder
