# Supplementary material for: A Qualitative Exploration of Ethical Aspects of Using AI in Parkinson Disease: Patient Panel Study
Source: JMIR AI. 2026 Apr 28;5:e74144. doi: 10.2196/74144 (PMC13123883; doi:10.2196/74144)
Supplement: Multimedia Appendix 1 [file ai-v5-e74144-s001.docx]

Interview Guide: Participation in PD Patient Panel – AI PROGNOSIS

[*Brief explanation about purpose of interview, that is will be recorded, etc*.]

**1. Personal background:**

1.1 Background

- Tell us a bit about your background. E.g. age, occupation

**2. PD Background:**

2.1 Diagnosis timing

When were you diagnosed with Parkinson's disease?

2.2 Diagnosis process

- Could you briefly describe the diagnosis process?
- What steps were involved, and how did you experience it?

**3. Experiences with Participation in Research and/or Patient Panels:**

3.1 Previous participation

- Do you have any prior experiences participating in research projects or patient panels?

3.2 Experiences

- If so, could you share your experiences and what you found most meaningful or challenging?

**4. Expectations and/or concerns with Participation in this Project:**

4.1 Expectations

- What expectations, if any, do you have about participating in this specific research project?

4.2 Concerns

- What concerns, if any, do you have about participating in this specific research project?

4.3 Hopes/Goals

- What do you hope to achieve by participating in this project?

**5. View on Prediction Models Generally:**

[*Brief introduction of the work to be done in AI-PROGNOSIS incl. the 3 planned prediction models (PD diagnosis, disease progression, and prediction of medication effect*)]

**5.1 General interest**

- How do you view prediction models in general within healthcare and research?

5.2 Advantages and challenges

- What do you see as the specific advantages and challenges of using prediction models?

**6. View on Prediction Models planned in AI-PROGNOSIS**

6.1 General perspective

- What is your perspective on the use of prediction models to predict Parkinson's disease diagnosis, disease progression, and medication effects?

6.2 Specific advantages and challenges

- Are there specific advantages or challenges you see when it comes to using prediction models for PD diagnosis, disease progression, and medication effect?

**7. Closing Questions:**

7.1 Participation and significance

- How do you perceive your own involvement in the research process, and how important do you think patients' perspectives are in such projects?

7.2 Any additional wishes

- Do you have any additional wishes or things you would like to highlight in connection with your participation in this research project?
