# Supplementary material for: A Qualitative Exploration of Ethical Aspects of Using AI in Parkinson Disease: Patient Panel Study
Source: JMIR AI. 2026 Apr 28;5:e74144. doi: 10.2196/74144 (PMC13123883; doi:10.2196/74144)
Supplement: Multimedia Appendix 2 [file ai-v5-e74144-s002.pdf]

ai·prognosis

Focus group on user needs

PwP focus group 2

22 February 2024

Funded by the European Union

First things first

- Research participant information was sent out ahead of the meeting
- Questions?
- Record consent

Workshop agenda

Break when needed

1. Introductions
2. Workshop targets
3. Definitions & assumptions
4. The AI-PROGNOSIS tools
5. Discuss high-level product features and user needs (in Miro)
6. Next steps

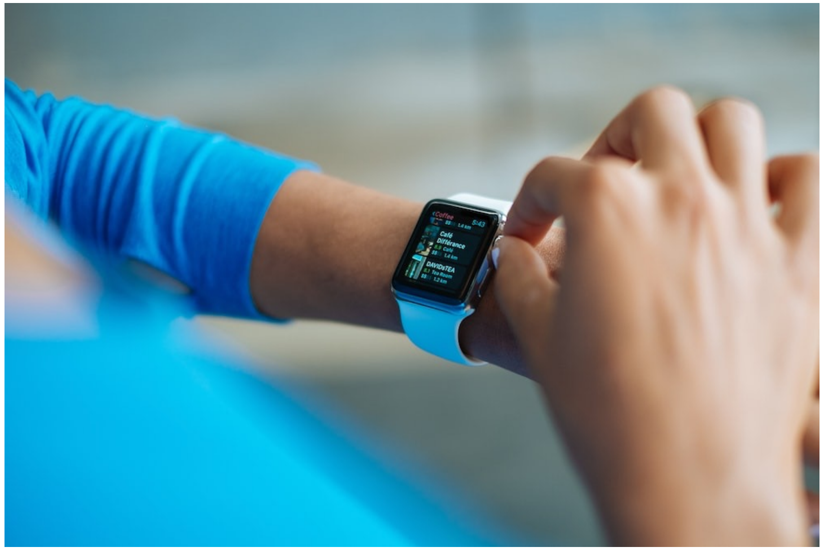

Introductions

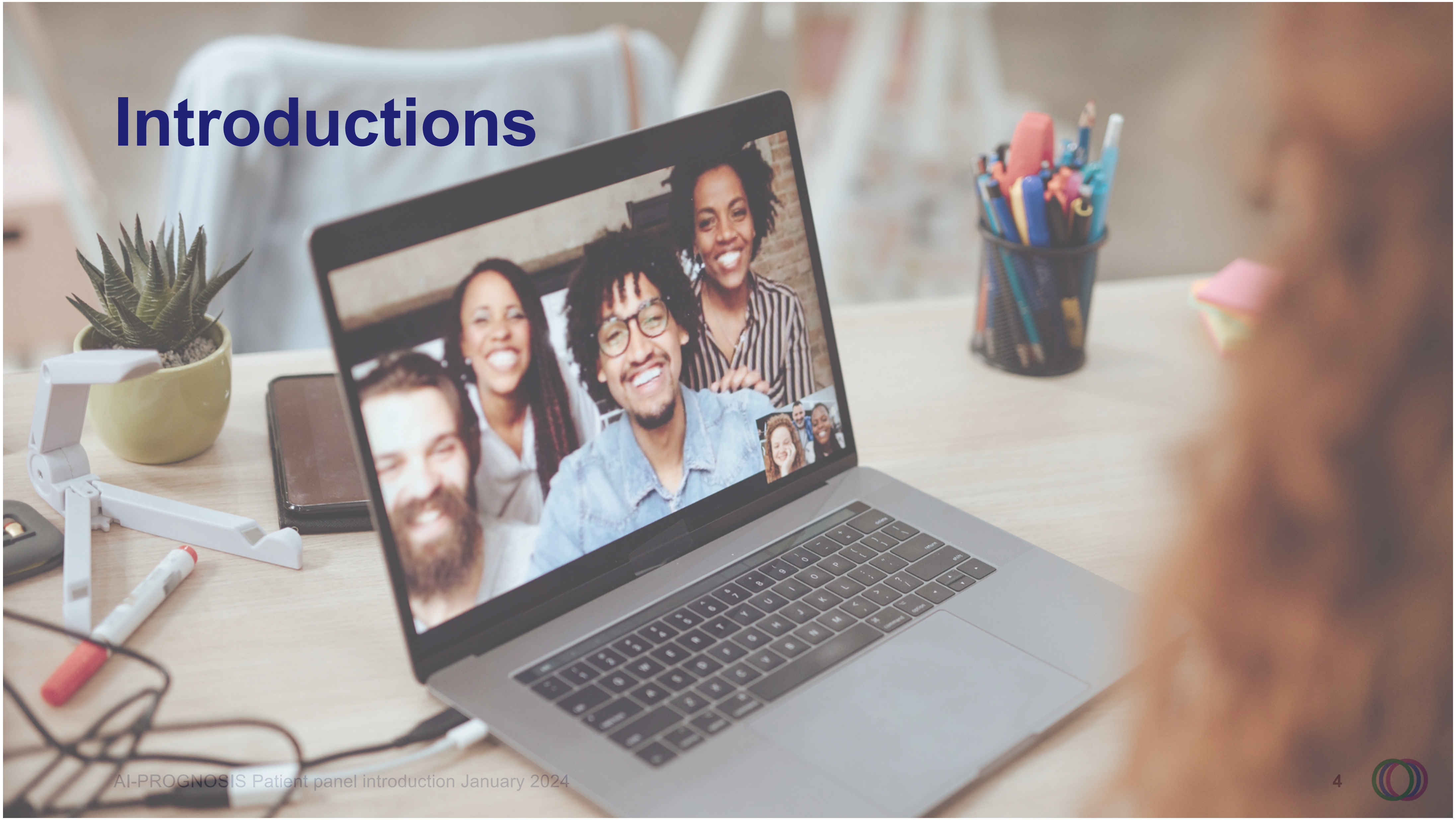

Workshop targets

- Get an overview of app features
- Identify and prioritise user needs
- If there is time…
  - Identify and discuss potential user challenges

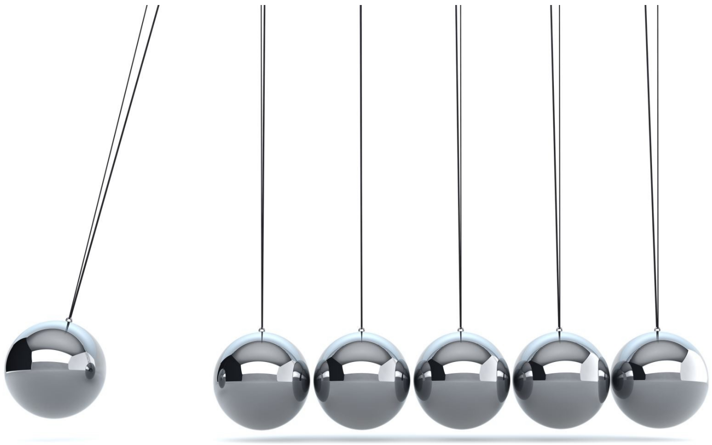

Definitions 1(2)

High-level features

- will deliver value to the end users of the AI-PROGNOSIS apps.
- Examples:
  - mAI-Health - users (persons without PD) will be able to track their risk of Parkinson's disease
  - mAI-Care - users (PwP) will be able to track disease progression
  - mAI-Insights - users (secondary care professionals) will be able to see projections of their attending patients' progression.

User needs or user requirements

- functions that the users expect/want the application to support.
- Examples:
  - The mAI-Health app users should be able to see metrics about their risk of getting PD. (Priority: Low)
  - The mAI-Care app users should be able to view when the next tasks/tests must be performed. (Priority: High).

Definitions 2(2)

Functional requirements

- product features or functions that developers must implement to enable users to accomplish their tasks.
- Examples:
  - The mAI-Health app must allow the user to perform a memory test (Priority: High).
  - The mAI-Care app should allow the user to choose what time of the day they receive their unperformed task reminders (Priority: Medium).

Non-functional requirements

- system qualities and constraints. They will be more or less common across the three products.
- Examples
  - UI accessibility, e.g. color contrast, text size
  - system security, e.g., encrypted databases, data transmission over HTTPs.

Assumptions

- Process
  - Your input will have an impact
  - Focus will be on user needs
- Products
  - The AI-PROGNOSIS tools will be based on the ”study app”
  - The apps will be in both iOS & Android
  - The AI models will work perfectly

mAI- Health

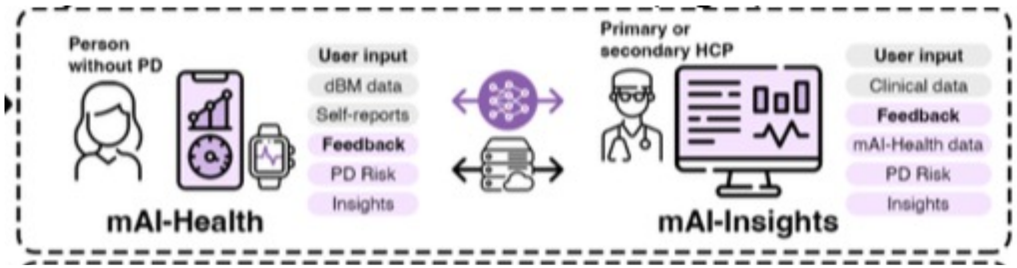

High level feature

User need

1.User Interaction:
- Users interact with the mAI-Health mobile app on their smartphones.

2.Data Collection:
- The app collects data from two primary sources:
  -SmartWatch-Tracked Data
  -Self-reports on Relevant Risk Factors/Symptoms

3.PD Risk Assessment Model:
- The collected data feeds into the PD Risk Assessment Model.
- The model calculates a quantitative PD risk score based on the input data.

4.Explainable Insights:
- The app provides explainable insights derived from the PD risk assessment.
- Users receive understandable information about their personalized risk of acquiring Parkinson's Disease.

5.User Interface:
- The app displays the results and insights in a user-friendly interface.

6.Data Visualization:
- Visual representation of data (charts, graphs)
- Clear presentation of the quantitative PD risk score and associated insights.

mAI- Care

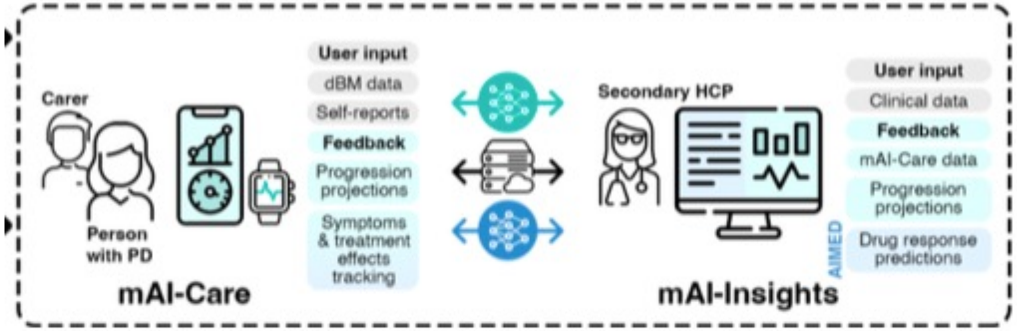

1.User Interaction:
- Persons with Parkinson's (PwP) and their caregivers interact with the mAI-Care mobile app on smartphones.

2.Data Collection:
- The app collects data from multiple sources:
  -SmartWatch-Tracked Data
  - Occasional Self-Reports on Symptoms and Condition
  - Clinical Data Shared by Attending Physician

3.PD Progression Predictive Model:
- The collected data feeds into the PD Progression Predictive Model.
- The model generates personalized projections of PD progression for the individual.

4.Symptom and Treatment Tracking:
- Users can input and track their symptoms in the app.
- Users can log information about their treatment, medication efficacy, and any side effects experienced.

5.User Interface:
- The app displays personalized projections of PD progression in an easy-to-understand format.
- Graphical representation of symptom trends and treatment effects for better visualization.

6.Clinical Data Integration:
- Integration with clinical data shared by the attending physician for a comprehensive overview of the individual's health.

7.Notification System:
- Implement a notification system to remind users to input self-reports and take medications.
- Caregivers may receive notifications or updates about the PwP's condition.

8.Data Security and Privacy:
- Ensure robust security measures to protect sensitive health data.

Miro
