# Supplementary material for: A Qualitative Exploration of Ethical Aspects of Using AI in Parkinson Disease: Patient Panel Study
Source: JMIR AI. 2026 Apr 28;5:e74144. doi: 10.2196/74144 (PMC13123883; doi:10.2196/74144)
Supplement: Multimedia Appendix 3 [file ai-v5-e74144-s003.docx]

Focus group guide

On-boarding process

1. Envision the journey through registering and onboarding the app. What would you like or not like from such a toolkit?

mAI-Health tool for persons at risk

1. What are the main user-needs…?
   1. When it comes to user authentication?
   2. When it comes to data collection?
      1. Think of any concerns you might have relating to a smartphone collecting data from a smartwatch, regarding different signs and symptoms
      2. What would be a reasonable frequency and time to spend on self-reported data? Would gamification features help you to keep tracking?
   3. When it comes to data presentation and use?
2. If you were to imagine such a thing would have been available before your diagnosis, what are your thoughts on this PD risk assessment model for people that are not yet diagnosed?
3. What would have been helpful for you in that situation?
4. *When* would it have been helpful to have been suggested to collect this data? Would it have been at the first visit during the evaluation process?
5. What advantages/possibilities do you see?
6. What risks/challenges do you see?
7. What are your thoughts on presenting the risk score? To whom and why?

mAI-Care tool for PwP

1. What are the main user-needs…?
   1. When it comes to user authentication?
   2. When it comes to data collection?
      1. Think of any concerns you might have relating to a smartphone collecting data from a smartwatch, regarding different signs and symptoms
      2. What would be a reasonable frequency and time to spend on self-reported data?
      3. Would gamification features help you to keep tracking?
   3. When it comes to data presentation and use?
2. Thoughts on the prediction of progression?
   1. How relevant would such prognostic data be for you in your life?
   2. Do you want to know this?
3. Thoughts on the prediction of medication response?
4. Thoughts on informal carers (family, spouse) having access to such an app?
5. Thoughts on the use of clinical data for the predictions?
