# Supplementary material for: A Qualitative Exploration of Ethical Aspects of Using AI in Parkinson Disease: Patient Panel Study
Source: JMIR AI. 2026 Apr 28;5:e74144. doi: 10.2196/74144 (PMC13123883; doi:10.2196/74144)
Supplement: Multimedia Appendix 4 [file ai-v5-e74144-s004.docx]

Appendix 4. Demonstration of analysis process.

| **Raw data** | **Code(s)** | **Sub-theme** | **Theme (Principle)** |
| --- | --- | --- | --- |
| *“the [medication response prediction] is the interesting part of the program. I think it could be interesting as I have had the disease for many years. And you don't know what's really happened“* (PwP5).  *“it's very important, I think, to define all these possible earliest symptoms and discuss how you can measure those as seamlessly as possible, with as little friction for the user”* (FG01). | interested in prognosis and med response. Many questions about own PD over time    would have liked an AI toolkit pre-diagnosis to identify their first symptoms and in what order | AI could increase one’s PD knowledge and understanding | Autonomy |
| *“in this case was you you also had this decision support system am I am I inside because I think you should analyze all everything that the specialists are actually doing and see how much of that could be automated or eliminated or improved by having smart digital services and having patients that are encouraged and given the tools to be more active themselves, so they can lessen the work burden on the doctor and not having to do this routine visits“* (PwP03).  “*I think [AI] can be advantageous. Yes, it's lots of data to be processed and only computers are able to process a huge amount and to try to find patterns out of it. And maybe to tell you, what we have, we have some level of risk aversion of confidence that you might be affected by this frenzy, so that this treatment would be better for you, whatever*” (PwP13). | patients can be more active through digital tools/services and thus lessen the burden on the HCP            AI can find patterns that humans cannot and use for treatment optimization | AI could support HCPs with improved and timely care | Beneficence |
| *“It’s a tough topic it's really touchy to to know whether you can talk to someone and tell him only there is risk that you that you are affected with this and you don't know you don't know in advance of the person who will react some could be very depressed some could say okay, what can I do to reduce the risk etc. So, yeah, that's a psychologically tough topic and I think you would need…it's difficult to predict how people would react”* (PwP13).  *“Now the question with Parkinson's is we don't really know, substantively other than exercise, that anything can massively change at the moment, but they might do for the future. So that for me, this is where it's always almost for me, this should be number one, before we even determine whether we even do it. Because I think you have to be quite, very considerate of the consequences, and I think the worst consequences are that you might tell someone who has Parkinson's, who takes it badly. Who could you know, kill themselves or something. You know, like, we I didn't know about you, but I felt like that when I was first diagnosed, I wouldn't let myself walk near a cliff. It's just, you know, life changing. So I think there are some very delicate, nuanced issues that need to be teased out of that”* (FG01). | conveying risk of PD is a touchy topic; you don't know how the person will react; risk of depression etc. | Potential harm of knowing | Non- maleficence |
| *"I'm convinced the reason I'm doing semi-okay after 12 years [with PD] is, among other things, lifestyle, exercise, nutrition, and lack of stress. But I'm also acutely aware that that's not a solution for a young father with three kids and a full-time job, or somebody living in Uganda, or, yeah, most of this plan is in fact, saying I do yoga every morning and try to cut down on stress. It's very much a first-world solution to the problem. Though, finding universally applicable ways of slowing progression would be a personal priority of mine"* (PwP04). | equity problem, not everyone can implement optimal lifestyle if recommended to | Fair and equitable use of AI | Justice |
| *“I find myself impressed and somewhat uncomfortable, I suppose is the right word, by the evidence connection between you and the machine and its presentation so far as the use of it in the real world. I think the media are already guilty of this. There is the simplistic connection between AI and sorting out complicated stuff. Now, any system, any bag of information is only as good as the information that's provided. And if we chat rubbish into the model, we will get we will not only get rubbish conclusions. If not, we will not understand how we got to those conclusions. And if we go away and do stuff, and we go into what it suggests, the law of unintended consequences will, will operate. So I am indeed, I will have to be convinced about the quality of the data going into the model. Before I can accept any output from it*” (PwP10). | needs to be convinced that data it is being fed is of high quality | Transparency and reliability of AI | Trust, truthfulness and confidentiality |
